# Attitudes towards the dual health insurance system and inequalities in health care in Germany – results of a population survey

**DOI:** 10.1186/s12913-025-12847-x

**Published:** 2025-05-14

**Authors:** Olaf von dem Knesebeck, Jens Klein

**Affiliations:** Institute for Medical Sociology, University Medical Center Hamburg-Eppendorf, Martinistr. 52, Hamburg, 20246 Germany

**Keywords:** Attitudes, Population survey, Statutory health insurance, Private health insurance, Health care inequalities

## Abstract

**Background:**

In Germany, type of health insurance is an important aspect of health care inequalities because there is a dual structure of statutory and private health insurance and there are incentives for a preferential treatment of privately insured patients. Two questions will be addressed in the study: What are the public attitudes towards the dual system of health insurance and inequalities in health care between those with statutory and private insurance? Are these attitudes associated with socio-demographic characteristics, health insurance and political party preferences?

**Methods:**

Based on a random sample, an online survey among the adult population (18 + years) in Germany was conducted (*N* = 2,201). Attitudes towards statutory and private health insurance were assessed using four items. Two items related to inequalities in access and quality of care; two were aimed at the system structure and remuneration. Age, gender, education, migration history, region of residence, health insurance and political party preference were included in the analyses as predictors.

**Results:**

About 77% of respondents agreed with the statement that all insured persons in Germany have the same access to medically necessary care. Women were less convinced in this respect. Just over 20% agreed with the statement that people with statutory and private health insurance receive the same quality of medical care. Agreement was particularly high among men, older people and those with private insurance. Almost 80% were in favor of abolishing the coexistence of statutory and private health insurance. Agreement was lower among privately insured people and supporters of the liberal party; however, even in these subgroups, a majority of over 60% were in favor of abolition. Just over 15% found the different remuneration of services for those with statutory and private insurance acceptable. Agreement was significantly higher among older people and those with private insurance.

**Conclusions:**

A clear majority of the German population perceives inequalities in the quality of medical care between those with statutory or private health insurance and is in favor of abolishing the dual system of statutory and private health insurance. These majorities can be found in all socio-demographic subgroups and across all political party lines.

**Supplementary Information:**

The online version contains supplementary material available at 10.1186/s12913-025-12847-x.

## Background

Health care in Germany is characterized by a dual system of statutory health insurance (SHI) and private health insurance (PHI). While health insurance generally is compulsory for everyone living in Germany, the conditions for admission to one or the other type of health insurance are linked to employment status, occupational group and income level of a person, amongst others [1]. Accordingly, only people with an income over a certain limit, self-employed, and public servants can choose a PHI [2]. Currently, around 11% of the population are privately insured and 89% have a SHI [3]. Germany is the only country in Europe with a dual health insurance system. In the SHI, type and scope of medical services covered by the insurance are regulated by law, whereas available services depend on the tariff in the PHI. Contributions in the SHI are related to the income of the insurant. In the PHI, contributions depend on the age at entry and health risks of the insurant as well on the scope of benefits. While only few physicians solely treat privately insured patients, they receive different levels of remuneration for treating patients with PHI or SHI. For privately insured patients, doctors can increase the price for medical treatment by a mark-up factor (usually a 2.3-fold increase is used). These differences in physician reimbursement rates create incentives for the preferential treatment of privately insured patients and can thus lead to inequalities in health care [4, 5].

Inequalities in health care can relate to access, utilization and quality [6]. According to Saurman [7], access to health care is a multidimensional concept describing the fit between the patient and the health care system. Health care utilization is a behavior of individuals that is influenced by needs, preferences, and opportunities. Quality of care can be classified under the categories structure, process, and outcome [8].

Empirical studies documented that there are inequalities in health care between people with statutory and private insurance in Germany [2]. However, the results vary depending on the area of health care (e.g. outpatient care, inpatient care, prevention, long-term care) and aspect of care (access, utilization, quality). With regard to access, for example, there were differences in waiting times [9–12] and in the accessibility of medical care services [13] to the disadvantage of people with SHI. Overall, SHI patients used outpatient medical services more frequently. Moreover, there were differences in consulting general practitioners or specialists; the latter were consulted more frequently by patients with a PHI [14]. In terms of process quality, patients with a SHI rated quality of communication with specialists worse than privately insured patients [12, 15]. In contrast, there were no consistent differences between PHI and SHI patients for outcome quality indicators (e.g. quality of life, survival rates) [16, 17]. However, evidence on inequalities in health outcomes related to insurance is not conclusive as respective studies are scarce.

The coexistence of SHI and PHI has long been the subject of controversial debate in Germany [18, 19]. However, there are only a few studies that take a differentiated look at the population’s attitudes towards the SHI-PHI system. Böcken and Altenhöner [20] analyzed attitudes towards solidarity in health insurance, focusing on the differences between respondents with SHI and PHI. In a recent study [19], people with SHI and PHI were asked about the solidarity principle and reform options in health insurance. However, overall, there is a lack of studies that analyze such attitudes in a differentiated manner for different population groups. Against this background, the present study addresses two questions: What are the public attitudes towards the dual system of health insurance and inequalities in health care between those with SHI and PHI? To what extent are these attitudes associated with socio-demographic characteristics, health insurance and political party preferences?

## Methods

### Study design and sample

The analyses are based on an online cross-sectional survey carried out by a social research institute (forsa) in November and December 2022 [12]. A random sample of the adult population (aged 18 and over) was drawn from a panel recruited by telephone. A dual-frame approach was used, which included both landline and mobile phone numbers. The panel is a population-based, representative sample of adults living in Germany. It is updated regularly and consisted of around 120,000 people. The participants are regularly interviewed regarding various topics. A sample of 5,619 people who stated that they use the internet was randomly selected from the panel and invited to take part in the survey by email. After three reminders, *N* = 2,201 people took part. The aim was a sample size allowing analyses of differences in attitudes between respondents with SHI and PHI, assuming that a proportion of around 10% of respondents were privately insured. The sample was weighted according to age, gender, federal state and education in accordance with official German statistics [21], so that it adequately represented the adult population in Germany with regard to these socio-demographic characteristics. The study was approved by the local psychological ethics committee at the Center for Psychosocial Medicine of the University Medical Center Hamburg-Eppendorf (No. LPEK-0563).

### Survey instruments

Attitudes towards SHI and PHI were surveyed using four items. Two items related to possible inequalities regarding access to and quality of health care: “People with statutory or private health insurance receive the same quality of medical care.” “All insured persons have access to medically necessary health care.” Two further items focused on the system structure and remuneration: “The coexistence of statutory and private health insurance should be abolished.” “It is acceptable that a doctor is paid differently for the same medical treatment, depending on whether the patient has private or statutory insurance.” All items had a four-point response scale (“fully agree”, “somewhat agree”, “somewhat disagree”, “fully disagree” and furthermore, “don’t know”).

The socio-demographic characteristics gender (male, female), age (three groups: 18–40 years, 41–59 years, 60 years and older), migration history (three groups: no migration history, 1st generation migrant, 2nd generation migrant), education (three groups based on the CASMIN classification [22]: low, medium, high) and region of residence (Western, Eastern Germany) were taken into account as predictors. Health insurance (SHI, PHI) and political party preference were also included in the analyses. The latter was determined by the question “Is there a political party that you are closer to than others? Which one?“. The following answer options were available: CDU (Christian Democratic Union), SPD (Social Democratic Party), Die Grünen (Green Party), FDP (Free Democratic Party), AfD (Alternative for Germany), Die Linke (The Left Party), other, no political party, and no answer. While the CDU is considered a conservative party, the AfD is a far right populist party. An excerpt from the questionnaire including all measures described in these paragraphs can be found in the supplementary file.

### Analyses

First, frequencies of the four items measuring attitudes towards SHI and PHI were analyzed. In order to explore associations with socio-demographic characteristics, health insurance and political party preferences, cross tabulations and Chi^2^ tests were used. Subsequently, logistic regression analyses were carried out in which the predictors were introduced simultaneously. For these analyses, the four items were dichotomized, with agreement (fully agree/somewhat agree) set to 1 in each case. The analyses were carried out with SPSS 29 [23].

## Results

Sample characteristics regarding sociodemographics, health insurance, and political party preference are documented in Table 1. Table 2 shows the distribution of the four attitude items: 21.2% of the respondents fully or somewhat agreed with the statement that people with statutory or private health insurance receive the same quality of medical care. A clear majority of 77.2% agreed that all insured persons have access to medically necessary care. Almost 79% of respondents were in favor of abolishing the coexistence of SHI and PHI and 15.7% thought it was acceptable for a doctor to be paid differently for the same medical treatment, depending on whether the patient has a private or a statutory insurance. In the following analyses, the “don’t know” answers were excluded.

**Table 1:** Sample characteristics (*N*=2,201)*

	n (%)
Gender (0)	
Female	1,124 (51.1)
Male	1,077 (48.9)
Age (0)	
18-40 years	721 (32.7)
41-59 years	743 (33.7)
60 years +	737 (33.5)
History of migration (37)	
No	1,670 (77.2)
Yes, 1^st^ generation	159 (7.4)
Yes, 2^nd^ generation	335 (15.5)
Education (64)	
Low	657 (30.7)
Middle	655 (30.6)
High	825 (38.6)
Region (0)	
Eastern Germany	331 (15.0)
Western Germany	1,870 (85.0)
Health insurance (8)	
Statutory	1,921 (87.6)
Private	272 (12.4)
Political party preference (138)	
CDU	394 (19.1)
SPD	331 (16.0)
Green Party	416 (20.1)
FDP	100 (4.9)
AfD	138 (6.7)
The Left Party	106 (5.1)
Others	59 (2.9)
None	519 (25.5)

*Weighted data; number of missing cases in brackets

**Table 2:** Distribution of attitudes towards the dual health insurance system (*N* = 2,201, %)

	Fully agree	Somewhat agree	Somewhat disagree	Fully disagree	Do not know
"People with statutory or private health insurance receive the same quality of medical care.“	5.2	16.0	42.1	30.9	5.8
"All insured persons have access to medically necessary health care.“	30.9	46.3	15.9	3.6	3.3
"The coexistence of statutory and private health insurance should be abolished.“	53.1	25.7	8.5	5.6	7.1
"It is acceptable that a doctor is paid differently for the same medical treatment, depending on whether the patient has private or statutory insurance.“	3.7	12.0	30.7	49.1	4.6

Men, older respondents, first-generation migrants, respondents from Eastern Germany and privately insured respondents significantly more often agreed with the statement that people with statutory and private insurance receive the same quality of medical care (Table 3). Political party preference was also significantly associated with this statement, with highest levels of agreement among supporters of the CDU, SPD and The Left Party. Agreement with the statement that all insured persons have access to medically necessary care showed significant associations with gender, age and political party preference.

**Table 3:** Attitudes towards the dual health insurance system (fully/somewhat agree) according to socio-demographic characteristics, health insurance and political party preference (part 1)

	All insured persons receive the same quality		All insured persons have access to medically necessary health care.	
Characteristic (n)*	%	p**	%	p**
Gender		<0.001		<0.001
Female (1,124)	18.8		76.3	
Male (1,077)	26.4		83.4	
Age		<0.001		0.004
18-40 years (721)	14.6		80.1	
41-59 years (743)	21.2		76.1	
60 years +(737)	31.7		83.2	
History of migration		0.006		0.593
No (1,670)(1,670)	23.0		80.0	
Yes, 1^st^ generation (159)	30.5		82.6	
Yes, 2^nd^ generation (335)	17.6		78.6	
Education		0.595		0.784
Low (657)	23.9		79.5	
Middle (655)	22.6		80.8	
High (825)	21.6		79.4	
Region		0.007		0.052
Eastern Germany (331)	28.7		83.8	
Western Germany (1,870)	21.5		79.1	
Health insurance		<0.001		0.075
Statutory (1,921)	20.8		79.2	
Private (272)	35.0		83.9	
Political party preference		0.005		0.009
CDU(394)	27.2		85.8	
SPD (331)	28.9		80.8	
Green Party (416)	18.5		80.0	
FDP (100)	22.4		85.0	
AfD (138)	18.9		72.1	
The Left Party (106)	25.5		73.7	
Others (59)	18.6		74.1	
None (519)	19.5		79.6	

* Number of cases vary due to missing values, ** significance of Chi^2^-Test

Significantly higher approval ratings for the statement that the coexistence of SHI and PHI should be abolished were found among women, middle-aged respondents, respondents with a lower level of education, those with statutory health insurance and respondents from Eastern Germany (Table 4). Agreement was lower among respondents with a preference for the CDU (75.6%) and the FDP (63.8%). Different remuneration for patients with statutory or private health insurance was more often acceptable for men, older people, those with a lower level of education and those with private health insurance. Agreement was particularly low among respondents with a preference for the Green Party and those with no preference for a political party.

**Table 4:** Attitudes towards the dual health insurance system (fully/somewhat agree) according to socio-demographic characteristics, health insurance and political party preference (part 2)

	Abolish coexistence of statutory/private health insurance		Different remuneration for same medical treatment acceptable	
Attribute (n)*	%	P**	%	P**
Gender		<0.001		0.002
Female (1,124)	87.6		13.9	
Male (1,077)	81.8		19.0	
Age		0.027		<0.001
18-40 years (721)	84.2		11.8	
41-59 years (743)	87.6		12.6	
60 years + (737)	82.4		24.6	
History of migration		0.822		0.146
No (1,670)	84.7		17.0	
Yes, 1^st^ generation (159)	86.2		15.9	
Yes, 2^nd^ generation (335)	84.0		12.6	
Education		<0.001		0.026
Low (657)	88.3		19.7	
Middle (655)	86.2		15.2	
High (825)	80.8		14.7	
Region		0.028		0.089
Eastern Germany (331)	89.0		13.1	
Western Germany (1,870)	84.0		17.0	
Health insurance		<0.001		<0.001
Statutory (1,921)	88.2		14.8	
Private (272)	60.5		27.6	
Political party preference		<0.001		<0.001
CDU (394)	75.6		24.4	
SPD (331)	87.6		19.9	
Green Party (416)	88.5		10.4	
FDP (100)	63.8		16.5	
AfD (138)	84.5		19.7	
The Left Party (106)	93.0		14.9	
Others (59)	90.7		15.8	
None (519)	86.8		12.4	

* Number of cases vary due to missing values, ** significance of Chi^2^-Test

The multiple logistic regression showed that agreement with the statement that people with statutory and private insurance receive the same quality of medical care was significantly less likely among women, younger respondents and those with SHI, while positive associations were found amongst first-generation migrants and Eastern Germans (Table 5). Women and middle-aged groups were less likely to agree with the statement on access, while this was more often the case for Eastern Germans. The statement that the coexistence of SHI and PHI should be abolished was significantly associated with gender, age, health insurance and political party preference (CDU and FDP compared to SPD). The statement on remuneration was significantly associated with gender, age, region, health insurance and a political party preference for the CDU.

**Table 5:** Multiple logistic regression analyses: odds ratio, (95%-confidence interval), and significance

	All insured persons receive the same quality	All insured people have access to medically necessary health care	Abolish coexistence of statutory/private health insurance	Different remuneration for same medical treatment acceptable
Gender				
Female	0.66 (0.52-0.82)***	0.68 (0.54-0.86)**	1.45 (1.10-1.91)**	0.75 (0.58-0.97)*
Age				
18-40 years	0.41 (0.30-0.56)***	0.85 (0.62-1.19)	1.41 (0.98-2.02)	0.45 (0.32-0.64)***
41-59 years	0.70 (0.54-0.91)**	0.68 (0.51-0.91)*	1.52 (1.09-2.12)*	0.40 (0.30-0.55)***
History of migration				
Yes, 1^st^ generation	1.78 (1.20-2.63)**	1.34 (0.83-2.16)	1.18 (0.69-1.99)	0.86 (0.52-1.41)
Yes, 2^nd^ generation	0.90 (0.65-1.26)	1.08 (0.78-1.49)	0.99 (0.68-1.44)	0.85 (0.58-1.23)
Education				
Low	0.89 (0.66-1.21)	1.10 (0.80-1.52)	1.43 (0.99-2.07)	1.12 (0.79-1.58)
Middle	0.93 (0.69-1.25)	1.20 (0.89-1.62)	1.18 (0.84-1.67)	0.98 (0.70-1.37)
Region				
Eastern Germany	1.45 (1.05-2.00)*	1.44 (1.00-2.07)*	1.51 (0.98-2.33)	0.62 (0.41-0.93)*
Health insurance				
Statutory	0.54 (0.40-0.74)***	0.72 (0.49-1.05)	4.21 (3.05-5.81)***	0.50 (0.36-0.71)***
Political party preference				
CDU	0.87 (0.61-1.23)	1.45 (0.97-2.18)	0.42 (0.27-0.64)***	1.48 (1.01-2.16)*
Green Party	0.70 (0.48-1.01)	1.02 (0.69-1.49)	1.05 (0.64-1.71)	0.67 (0.43-1.05)
FDP	0.87 (0.49-1.54)	1.34 (0.71-2.53)	0.23 (0.13-0.42)***	1.12 (0.59-2.15)
AfD	0.68 (0.40-1.15)	0.67 (0.41-1.11)	0.58 (0.31-1.08)	1.60 (0.94-2.75)
The Left Party	1.04 (0.60-1.79)	0.66 (0.38-1.14)	1.50 (0.64-3.53)	1.10 (0.58-2.08)
None	0.77 (0.54-1.09)	0.98 (0.68-1.42)	0.74 (0.47-1.17)	0.88 (0.59-1.32)

Reference categories: male, 60 years +, no history of migration, high education, Western Germany, private health insurance, preference for SPD; other political parties were excluded; * *p*<0,05, ** *p*<0,01, *** *p*<0,001

## Discussion

A majority of around 77% of the respondents agreed with the statement that all insured persons in Germany have equal access to medically necessary care. Women in particular were less convinced in this respect. Only just over 20% agreed with the statement that people with statutory and private health insurance receive the same quality of medical care. Agreement was particularly high among men, older people and those privately insured. Almost 80% were in favor of abolishing the coexistence of statutory and private health insurance. The lowest level of support for abolition was found among privately insured people and supporters of the FDP. However, even in these subgroups, a majority of over 60% were in favor of abolition. Only just over 15% of respondents found the different remuneration of identical medical treatment for people with statutory and private insurance acceptable. Agreement was significantly higher among older people and those with a private insurance.

In the literature, access is seen as a multidimensional construct [7, 24]. Three important dimensions are “accessibility” (geographical accessibility), “accommodation” (organizational structure, e.g. waiting times) and “affordability” (financial viability, financial burden). Empirical studies indicate that people with a SHI have poorer access to health care services with regard to these dimensions compared to privately insured people [9–13]. Since the (health) relevance of such inequalities regarding access to medical services is often unclear, there is a discussion of limiting the equality claim to treatments that are “medically necessary” [25]. This was also taken up in the question included in our survey. Accordingly, more than ¾ of respondents assumed that there are no differences between the insurance types with regard to medically necessary treatment. The above-mentioned empirical studies hardly allow any conclusions to be drawn about disparities in access to medically necessary treatment. This is also due to the fact that the term “medical necessity” is not precisely defined and is, therefore, vague [26, 27].

A clear majority of 73% tended to assume that people with statutory or private insurance do not receive the same quality of medical care. There are now a number of empirical studies that deal with inequalities in process and outcome quality between persons with statutory and private insurance. These show that people with statutory insurance tend to rate process quality (e.g. with regard to communication with doctors) worse than those with private insurance [12, 15], while the findings on outcome quality (e.g. health-related quality of life, survival rates) are rather inconsistent [16, 17]. Overall, however, the study situation in this area is deficient.

According to our findings, there is a broad consensus among the population that the coexistence of SHI and PHI should be abolished. A survey conducted by Zok and Jacobs [19] of people with statutory and private insurance (in equal numbers) also showed that the dual insurance system met with less approval than the reform model, according to which the entire population should be insured under the statutory health insurance scheme (so-called “citizens’ insurance”). The advantages and disadvantages of the citizens’ insurance have repeatedly been the subject of controversial debate (e.g [18]). In the election manifesto of the SPD [28] and the Green Party [29], it was still stated as a political goal for 2021. However, the citizens’ insurance can no longer be found in the coalition agreement between the three current governing parties (SPD, Green Party, FDP) [30]. In our survey, a majority of people across all political party boundaries was in favor of abolishing the dual system. Still, there is a trend showing that agreement is lowest among respondents with a preference for the CDU and the FDP, while those in favor of the left party are the strongest supporters of abolishing the dual system.

The different reimbursement models of SHI and PHI also play a major role in the respective political discussion. Moreover, they contribute to inequalities in health care, as service providers can generate higher revenues with privately insured patients. Thus, there are incentives for unequal treatment. The present results show that almost 85% of the population do not consider this difference in remuneration to be acceptable. There is a consensus across all socio-demographic subgroups and political party boundaries in this regard.

With regard to the introduced predictors, it is noticeable that gender and age were significantly associated with all attitudes: Women and younger respondents were consistently more critical of the dual system. Health insurance also proved to be an important predictor; as expected, those with SHI were significantly more critical. In contrast, education and migration history played a rather subordinate role.

### Limitations

The analyses were based on an online survey. Although a random sample was drawn from a panel that was recruited offline, only people who use the internet were included. This can lead to a selection bias, as can the fact that only around 39.2% of those invited took part. To reduce potential bias, the data was weighted by age, gender, federal state and education according to official statistics using an iterative proportional method [31]. However, only people who can read German were included. This must be taken into account when interpreting the results on variations depending on migration history. In addition, only four items were used to measure attitudes towards the SHI-PHI system. These newly developed items cannot reflect the entire spectrum of attitudes. In terms of the socio-demographic characteristics, we only included education (and not income and/or occupational status) as an indicator of the social status. Finally, analyses concerning party preferences should be interpreted with caution as the number of cases is small for some parties.

## Conclusions

Health insurance is an important indicator of health care inequalities in Germany. A clear majority of the German population perceives disparities in the quality of medical care between people with statutory or private health insurance and is in favor of abolishing the dual SHI-PHI system. These majorities can be found in all socio-demographic subgroups and across all party lines. Such perceptions of inequalities and unfairness are relevant for health services research as they can affect trust in health care as well as utilization of health services [32].

## Supplementary Information

Supplementary Material 1.

## Data Availability

The dataset used is available from the corresponding author on reasonable request.

## Abbreviations

PHI: Private health insurance
SHI: Statutory health insurancemediation experience
