# Supplementary material for: Attitudes towards the dual health insurance system and inequalities in health care in Germany – results of a population survey
Source: BMC Health Serv Res. 2025 May 14;25:696. doi: 10.1186/s12913-025-12847-x (PMC12080041; doi:10.1186/s12913-025-12847-x)
Supplement: Supplementary file 1 — Supplementary Material 1. [file 12913_2025_12847_MOESM1_ESM.docx]

**Excerpt from the questionnaire**

|  | Please indicate whether you agree to the following statements regarding health care in Germany. | | | | | | |
| --- | --- | --- | --- | --- | --- | --- | --- |
|  |  | Fully  agree | | Somewhat agree | Somewhat disagree | Fully disagree | *Don’t know* |
|  | People with statutory or private health insurance receive the same quality of medical care. | ○ | | ○ | ○ | ○ | ○ |
|  | All insured persons have access to medically necessary health care. | ○ | | ○ | ○ | ○ | ○ |
|  | The coexistence of statutory and private health insurance should be abolished. | | ○ | ○ | ○ | ○ | ○ |
|  | It is acceptable that a doctor is paid differently for the same medical treatment, depending on whether the patient has private or statutory insurance. | | ○ | ○ | ○ | ○ | ○ |

|  | What is your gender? | | |
| --- | --- | --- | --- |
|  | ○ male | ○ female | ○ diverse |
|  | How old are you? | | ________ years |

|  | In which country were you born? | | | |
| --- | --- | --- | --- | --- |
|  | ○ in Germany | | ○ other country: _________________ *+ no answer* | |
|  | In which country was your mother/father born? | | | |
|  | Your mother: | ○ Germany | | ○ other country: ________________ |
|  | Your father: | ○ Germany | | ○ other country: ________________ |

| What is your highest educational degree? |
| --- |
| __________________________________________________________ |

|  | Do you have a private or a statutory health insurance? | | | | | |
| --- | --- | --- | --- | --- | --- | --- |
|  | ○ private | ○ statutory | ○ don’t know |  |  |  |

|  | What is the postal code of your place of residence? |  |
| --- | --- | --- |
|  | ________ |  |

|  | Is there a political party that you are closer to than others? Which one? | | | | |
| --- | --- | --- | --- | --- | --- |
|  | ○ CDU/CSU | ○ SPD | ○ Bündnis 90/Die Grünen | ○ FDP | ○ Die Linke |
|  | ○ AFD | ○ other: ___________________ | | ○ no party | ○ no answer |
